# Dietary citrulline supplementation enhances milk production in lactating dairy goats

**DOI:** 10.1186/s40104-025-01187-9

**Published:** 2025-04-05

**Authors:** Arianna N. Lopez, Makenzie G. Newton, Claire Stenhouse, Erin Connolly, Karina L. Hissen, Scott Horner, Guoyao Wu, William Foxworth, Fuller W. Bazer

**Affiliations:** 1 Departments of Animal Science, Texas A&M University, Kleberg Center, College Station, TX 77843-2471 USA; 2 Cooperative Extension Service, Prairie View A&M University, Prairie View, Texas, 77446 USA

**Keywords:** Arginine, Citrulline, Dairy goat, Lactation, Milk composition

## Abstract

**Background:**

Lactational performance depends heavily on age, health, and nutrition. L-Citrulline (Cit) is an effective precursor of L-arginine (Arg), an amino acid that has important roles in synthesis of nitric oxide (NO) and polyamines. Ruminal microbes degrade extracellular Arg; however, extracellular L-citrulline (Cit) is not degraded by ruminal microbes due to lack of uptake and can be fed unencapsulated as a precursor for Arg. As NO is a vasodilator, an increase in blood flow and transport of molecules to mammary tissue may enhance lactational performance and milk composition. Increases in polyamine production may increase milk protein synthesis within mammary tissue, thus increasing milk protein content. This study determined, for the first time, effects of dietary Cit supplementation on milk production and milk composition of Alpine dairy goats.

**Methods:**

Does were synchronized to estrus and bred to Alpine bucks. Parturition was induced on d 149 of gestation and does were suckled overnight allowing kid(s) to obtain colostrum before being milked 24 h later (d 1 of lactation). Does were assigned to either control (CON, *n* = 24) or Cit (CIT, *n* = 23) diets. The isonitrogenous control diet consisted of 97.63% basal diet and 2.37% supplement (1.37% L-alanine and 1.00% soybean hydrogenated oil). The CIT supplemented diet consisted of 97.63% basal diet and 2.37% supplement (0.5% Cit, 0.5% L-glutamine, 1% soybean hydrogenated oil, 0.37% cornstarch). Diets were group fed ad-libitum by treatment group. Blood samples were collected on d 0 and 30 of lactation, milk volumes measured twice daily, and on d 10, 20, and 40 of lactation, milk samples were collected.

**Results:**

CIT-treated does had greater daily milk production (*P* < 0.05) and there was an effect of day of lactation on daily milk production (*P* < 0.0001). Sire had significant effect on daily milk production as well (*P* < 0.05). Milk compositional analyses revealed Cit supplementation increased solid-non-fat (SNF; *P* < 0.05) and protein (*P* < 0.05) content in milk.

**Conclusions:**

Our novel results indicate that dietary supplementation of Cit fed ad-libitum in Alpine does increased daily milk yield, milk SNF content, and protein content. Supplemental Cit may be a proxy for Arg in goats to enhance lactational performance.

## Introduction

Lactational performance of dairy goats depends on multiple factors such as nutrition, breed, overall health, age, and environment. For Alpine dairy goats, the period of lactation can be more than 240 d [1]. Milk composition for Alpine dairy does averages 2.46%–3.44% fat, 2.79%–3.35% protein, 4.17%–4.3% lactose, and 7.5%–8.32% solid non-fat (SNF) [2, 3]. Of note, the consumption of goat milk is beneficial to individuals with allergens and gastro-intestinal disorders due to its lower content of lactose in comparison to cow’s milk [4]. As the dairy goat industry continues to grow globally, it is important to utilize management techniques to improve productivity. As new knowledge from research is available, it can be translated into management strategies in the dairy industry to improve productivity and profitability.

Nutritional status of females is a factor that greatly impacts milk production and composition, so it is imperative to optimize diets in order to optimize the quantity and quality of milk produced. In ruminants, extensive hydrolysis and fermentation of nutrients occur in the rumen by enzymes and microbes including bacteria [5]. Ruminants such as goats, cows, and sheep have a rumen, reticulum, omasum, and abomasum in comparison to the simple stomach in monogastric species [6]. The rumen is lined by papillae and inhabited by bacteria (95% of the microbial population), archaea, fungi, and protozoa [8]. These microbes have multiple roles in the digestion of proteins, degradation of amino acids (AAs), and protein synthesis [7, 8]. It has been suggested that AA uptake by ruminal microbes may be the limiting factor in protein metabolism in the rumen [9]. Once AAs are taken up by ruminal microbes, they are incorporated into protein or are broken down into ammonia for synthesis of AAs [7, 9–11]. In ruminant nutrition, a previous long-standing view is that all dietary AAs, including Arg and glutamine, undergo extensive degradation by ruminal microbes [12, 13]. However, it has been discovered that extracellular L-citrulline (Cit) is not degraded by the ruminal microbes of ruminants including sheep and cattle due to the lack of uptake by the microbes [14–16]. Therefore, Cit is a direct precursor for Arg in goats and can be directly supplemented in their diets without the need for encapsulation or protection to serve as a proxy for Arg. Endogenous Arg in ruminants is synthesized from AAs by ruminal bacteria and from Cit (formed from glutamine/glutamate and proline in enterocytes of the small intestine) by extrahepatic tissues [17]. Arg is a common substrate for the synthesis of nitric oxide (NO, the major vasodilator), polyamines (essential for DNA and protein syntheses), and creatine (essential for energy metabolism in skeletal muscle and other tissues) [13]. Thus, Arg supplementation improves reproductive and lactational performance in swine [18]. Unencapsulated Cit bypasses the rumen and enters the small intestine for absorption and extra-hepatic Arg synthesis in ruminants including goats, which may yield similar beneficial effects as for dietary Arg supplementation in pigs.

Based on the foregoing, we hypothesized that dietary Cit supplementation would increase the availability of Arg to improve milk production and milk composition in lactating dairy goats. This novel hypothesis was tested in the present study with Alpine dairy goats. Gaining insight into the impacts of dietary supplementation of Cit on lactational performance and milk composition is expected to suggest new management strategies to improve milk production and composition to benefit dairy goat and dairy cattle industries.

## Materials and methods

### Animals

Lactating Alpine dairy goats were utilized to study the effects of dietary supplementation of Cit (*n* = 47). All goats were fed to meet National Research Council requirements [19] and the composition of nutrients in the basal diet is summarized in Table 1. Cit was negligible in plant-sourced feedstuffs [5]. All goats were assigned randomly to either the control group (CON, *n* = 24), or the Cit group (CIT, *n* = 23). The isonitrogenous CON diet consisted of 97.63% basal ration and a 2.37% supplement (1.37% L-alanine and 1.00% soybean hydrogenated oil). The CIT diet consisted of 97.63% basal ration and a 2.37% supplement (0.5% Cit, 0.5% glutamine, 1% soybean hydrogenated oil, and 0.37% cornstarch). All does in each group were housed together and group fed with free access to feed and water throughout the study. The feed intake of goats was similar between the CON and Cit groups and was approximately 2.3 kg/d. In the Cit group, the intake of Cit was 11.5 g/d per goat.

**Table 1:** Composition of nutrients in the basal diet (as-fed basis)^a^

Nutrients	Content in diet
Dry matter	89.19%
Total digestible nutrients (TDN)	75.15%
Crude protein	18.13%
Lysine	0.91%
Methionine	0.26%
Crude fat	3.54%
Crude fiber	11.64%
Total calcium	0.89%
Total phosphorous (available P)	0.61% (0.27%)
Magnesium	0.30%
Sulfur	0.20%
Na	0.70%
Potassium	1.10%
Vitamin premix^b^	0.1%
Mineral premix^c^	0.1%
Acid detergent fiber (ADF)	13.04%
Neutral detergent fiber (NDF)	25.22%
Rumen-undegraded protein	6.01%
Rumen-degradable protein	12.12%

^a^Dietary ingredients were wheat middlings, corn, soybean hulls, soybean meal (48% crude protein), cottonseed meal, alfalfa, liquid binder, ground lime, sodium bicarbonate, soy oil, 1.52% salt mix, monocalcium phosphate (containing 21% phosphorus), mold inhibitor, and magnesium oxide

^b^The vitamin premix provided the following (IU/kg of the basal diet): vitamin A, 20,339; vitamin D, 4,458; and vitamin E, 66.3

^c^The mineral premix provided the following (mg/kg of the basal diet): manganese, 138.2; iron, 160.4; copper, 35.5; cobalt, 1.00; iodine, 2.08; zinc, 124.2; and selenium, 0.75

### Experimental design and sample collection

**Fig. 1:**
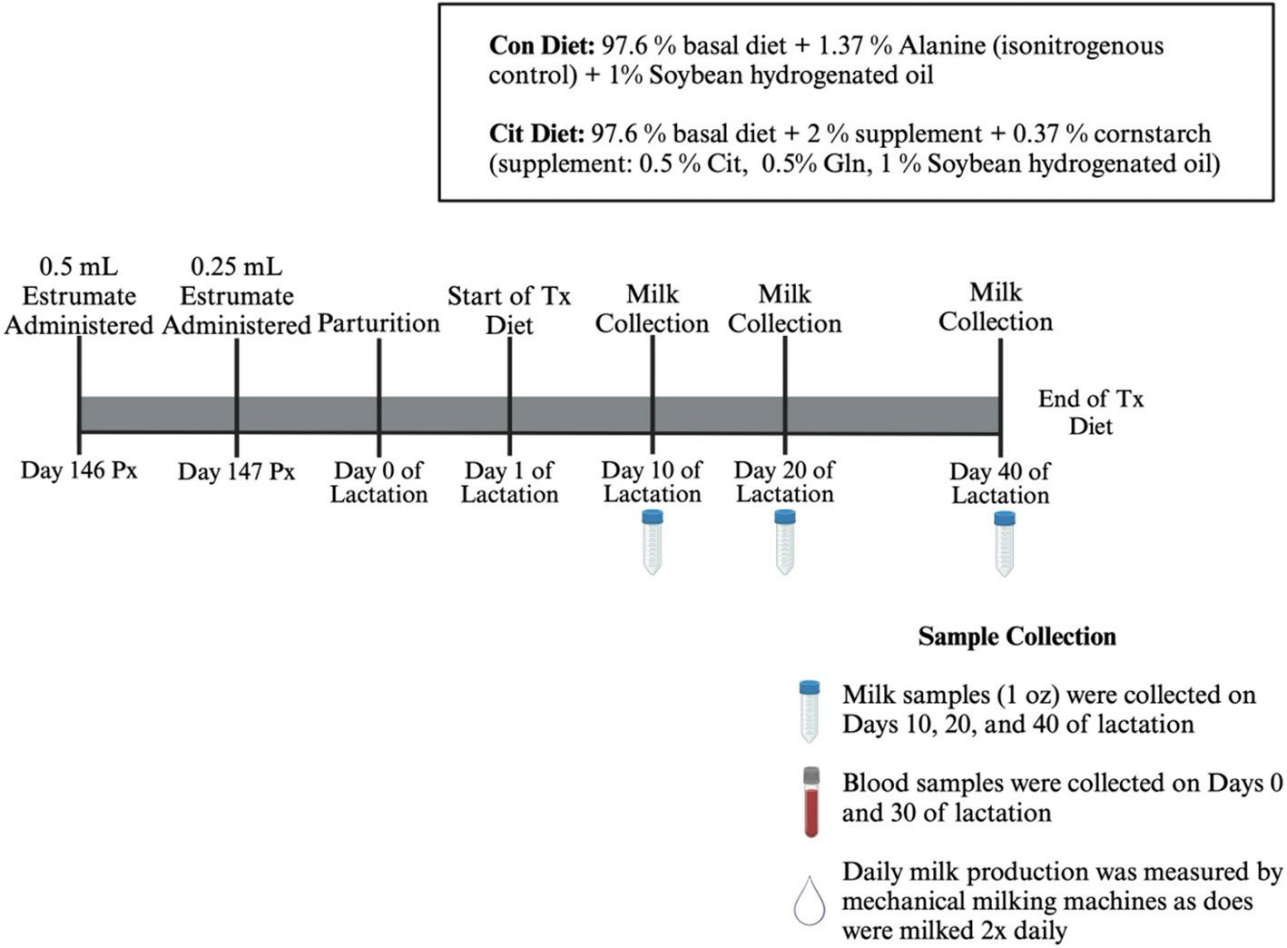
Outline of experimental design

Estrous cycles of reproductively mature Alpine dairy goats (*n* = 47) were synchronized by inserting a controlled intravaginal drug release device (CIDR; Zoetis, Parsippany, NJ, USA) and administering an intramuscular injection of GnRH (1 mL Factrel, Zoetis). All does then received an intramuscular injection of PGF2α (1 mL Estrumate, Merck, Rahway, NJ, USA) 10 d later. CIDRs were removed 2 d after the PGF2α injection and does were observed for estrus. The following day, the does were injected intramuscularly with GnRH (1 mL Factrel, Zoetis) and bred via natural service 20 h later to one of 13 sires. On d 146 of gestation, a single dose of 0.5 mL Estrumate (cloprostenol; Merck, Rahway, NJ, USA) was administered followed by a second dose of 0.25 mL Estrumate (cloprostenol; Merck, Rahway, NJ, USA) in the morning of d 147 to induce parturition on approximately d 149 of gestation. Following parturition (designated d 0 of lactation), does were allowed to nurse their kid(s) overnight to obtain colostrum. Each doe entered the milk line 24 h after kidding, which was designated d 1 of lactation. The randomly assigned control or Cit supplementation began on d 1 of lactation and continued through d 40 of lactation. All Alpine does were milked twice daily with mechanical milkers and the daily production of milk (L) recorded by DeLaval milk meters (Tumba, Sweden) for each doe at each milking. Volumes (L) of milk produced on d 0, 10, 20, and 40 were utilized to determine effects of Cit dietary supplementation on milk production. One ounce (29.6 mL) of milk was collected on d 10, 20, and 40 of lactation for compositional analyses. On d 0 and 30 of lactation, blood samples were collected in 10-mL BD vacutainer blood collection tubes from each doe via jugular collection, stored at 4 °C overnight. Blood samples were centrifuged (Eppendorf centrifuge 5920R Hamburg, Germany) at 5 °C for 18 min at 2,600 × *g*. Serum was harvested and stored at −20 °C until analyzed for concentrations of AAs using high pressure liquid chromatography (HPLC) analyses. Experimental design followed in this study is outlined in Fig. 1.

### Compositional analyses

Milk samples collected on d 10, 20, and 40 of lactation were allowed to cool to room temperature (25 °C) after collection. Once at room temperature, a somatic cell count was performed using a DeLaval DM SCC counter (DeLaval lnc., Tumba, Sweden). The raw milk samples were then analyzed using a Page and Pederson Lactometer (LactiCheck™-02 RapiRead, Page & Pedersen International Ltd., Hopkinton, MA, USA) for determination of butter fat, protein, lactose, and SNF.

### Analysis of amino acids

Effects of dietary supplementation of Cit on concentrations of amino acids in serum were determined using HPLC as described previously [20]. Briefly, each serum sample (100 µL) was acidified with 100 µL of 1.5 mol/L HClO_4_ and vortexed. The acidified sample was then neutralized with 50 µL of 2 mol/L K_2_CO_3_ and vortexed. The neutralized sample was centrifuged for 3 min at 10,000 × *g* in an Eppendorf centrifuge (Eppendorf centrifuge 5920R). The neutralized supernatant was then collected and diluted tenfold before analysis by HPLC using precolumn derivatization with *o*-phthaldialdehyde (OPA). The OPA reagent was prepared by dissolving 50 mg OPA in 1.25 mL HPLC-grade methanol, followed by 11.2 mL of sodium borate (pH 9.5), 50 µL of 2-mercaptoethanol, and 0.5 mL of Brij-23 (Sigma Aldrich, St. Louis, MO, USA). The assay mixture contained 1.4 mL of HPLC grade water (Fisher Scientific, Hampton, NH, USA), 100 µL of 1.2% benzoic acid (in 40 mmol/L sodium borate, pH 9.5), and 100 µL of sample. The assay mixture was derivatized in an autosampler (model 712 WISP, Waters, Milford, MA, USA) with 30 mmol/L OPA, and 15 µL of the derivatized mixture was injected into a Supelco 3-µm-reverse-phased C18 column (150 mm × 4.6 mm inner diameter, Sigma-Aldrich, St. Lois, MO, USA). Amino acids were separated by a solvent gradient comprised of Solution A (0.1 mol/L sodium acetate, 9% methanol, and 0.5% tetrahydrofuran, pH 7.2) and Solution B (100% methanol). Concentrations of amino acids in the serum samples were quantified relative to authentic standards using Millennium-32 Software (Waters, Milford, MA, USA).

### Statistical analyses

Statistical analyses of data using ANOVA for (a) a repeated-measures model [21] for serum amino acid concentrations on d 0 and d 30 and milk production on d 1 to 40, (b) one-way ANOVA for the effect of sire on milk production, and (c) the unpaired *t*-test for other variables were performed using JMP (Version JMP Pro 16). The normality of the distribution of daily milk production throughout the lactation curve was assessed using a goodness-of-fit test, and *P* $<$ 0.05 indicated that the data were not normally distributed. For data that were not normally distributed, a nonparametric Wilcoxon/Kruskal-Wallis test was used for mean comparisons. Pair-wise comparisons of means for data that were not normally distributed were made using the non-parametric Wilcoxon each-pair comparison test. The mean daily milk production data were normally distributed (*P* > 0.05). To compare means on a pair-wise comparison basis for data normally distributed, the each-pair student’s *t*-test was used. Least squares regression analyses were completed to determine any interactions among treatment, day of lactation, litter size, number of parities, and sire on daily mean milk production. Normality of mean daily milk production from each doe was assessed using the goodness-of-fit test. The normality of concentrations of AAs in serum samples collected on d 0 and 30 of lactation were assessed using goodness-of-fit tests. The concentrations of AAs were normalized to values from d 0 of lactation for each doe which was a baseline and factor of 1 to which concentrations of AAs in serum on d 30 of lactation were compared (concentration of AAs on d 30/concentration of AAs on d 0). A univariate repeated measures test was used to analyze those data to determine the effect of treatment over the 30-d period of lactation. A univariate repeated measures test accounts for day of supplementation while capturing correlations of AA concentrations within each doe [21]. This method considers d 0 of lactation baseline concentrations as a covariate and uses the difference from baseline as a method to normalize data [21]. In ANOVA analysis, post-hoc comparisons of means among treatment groups were made using the Student-Newman-Keuls test [21]. In all methods of statistical analyses, results were considered statistically significant at *P* $<$ 0.05 and trending towards significance at 0.05 $\leq$ *P* $<$ 0.10, and not significant at *P* ≥ 0.10.

## Results

### Effects of dietary Cit supplementation and sire on milk yield

**Fig. 2:**
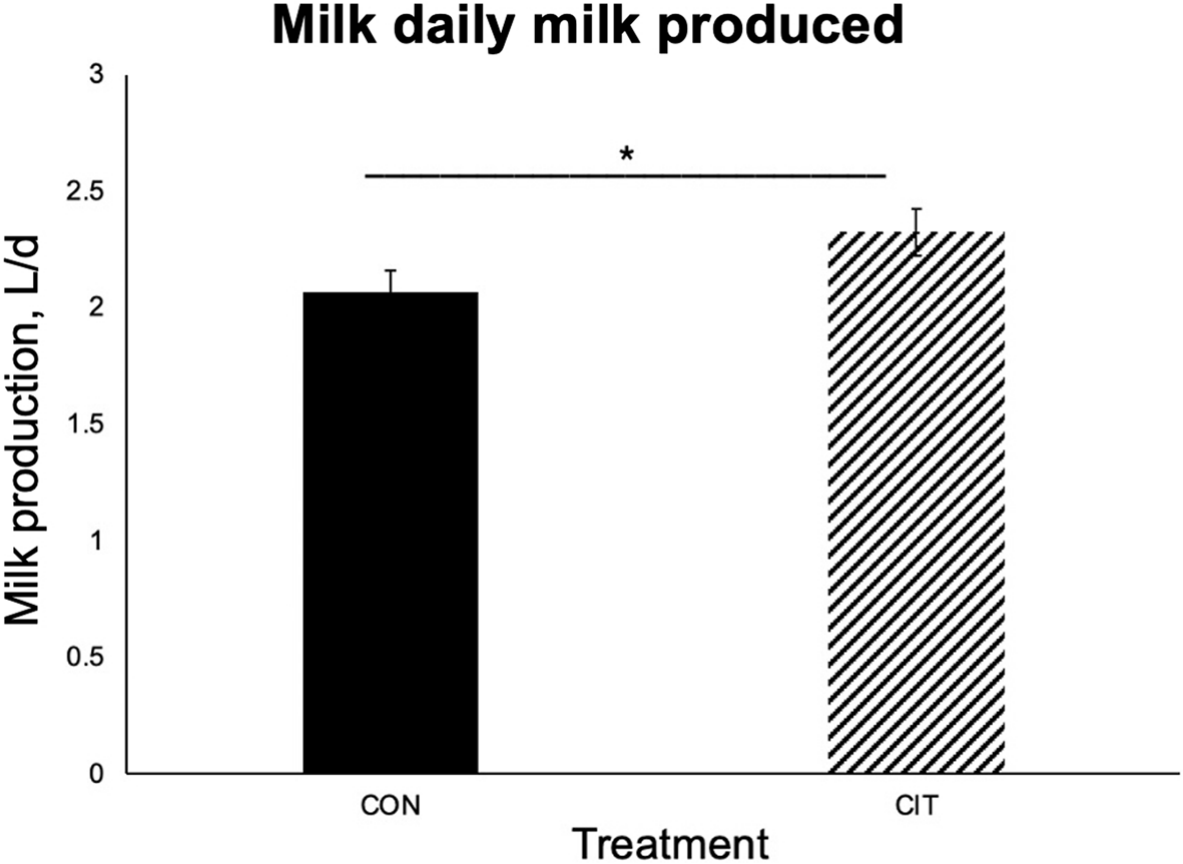
Daily milk production (L/d) differed between CON and CIT treatment groups. CIT does (*n* = 23) had greater (*P* < 0.05) daily milk production than CON does (*n* = 24). Data were analyzed using the unpaired *t*-test. Asterisks indicate that means differ from one another (*P < 0.05). Error bars represent SEM

**Fig. 3:**
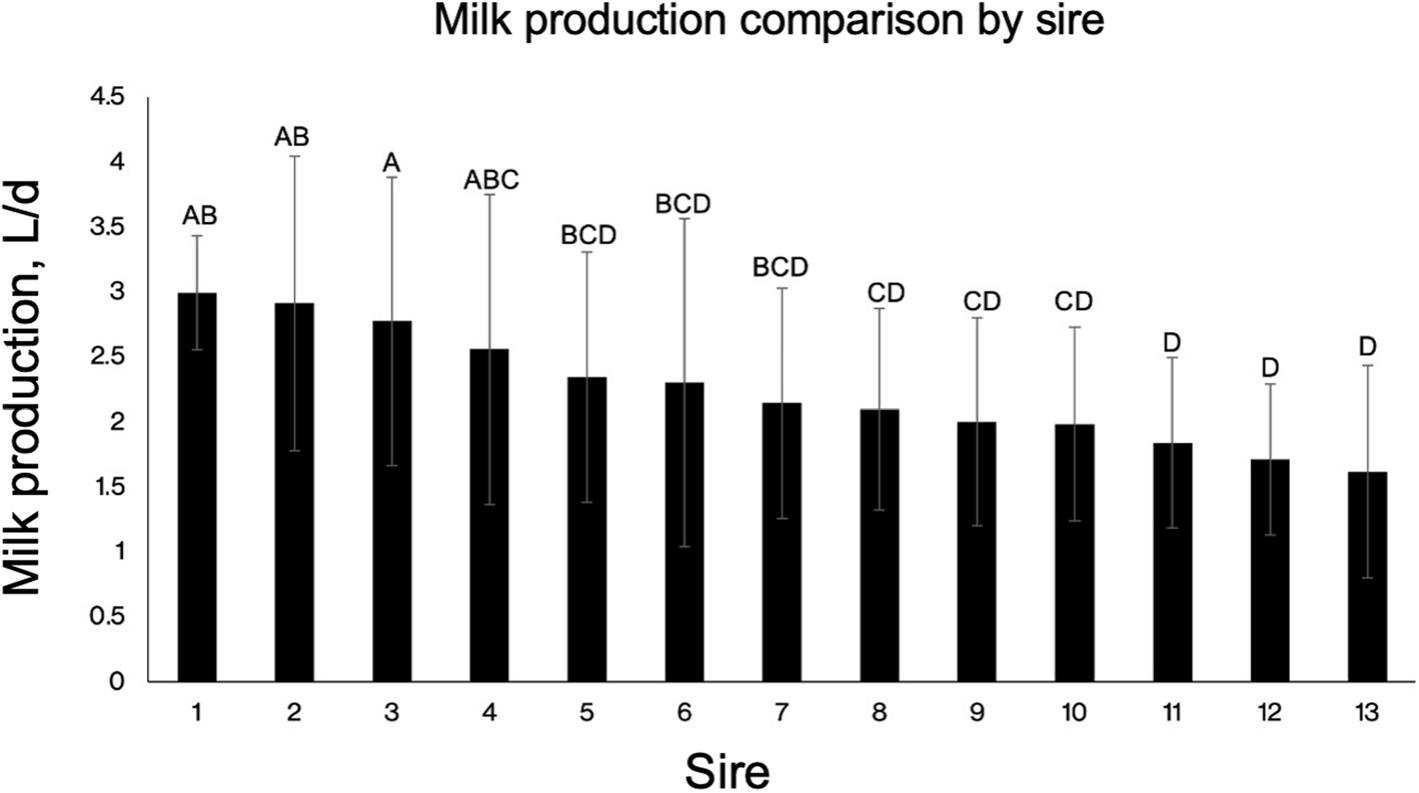
Effects of sire on daily milk production (L/d) by lactating goats. Values are mean ± SEM and statistical significance is shown by differing letters between sires based off pairwise comparison. Analyses of daily milk production by does bred to each sire indicated that sire affected daily milk production of does (*P* < 0.001). Note that significant differences in daily milk production are indicated by differing letters. (Sire 1, *n* = 2; Sire 2, *n* = 2; Sire 3, *n* = 1; Sire 4, *n* = 4; Sire, 5 *n* = 4; Sire 6, *n* = 6; Sire 7, *n* = 3; Sire 8, *n* = 5; Sire 9, *n* = 5; Sire 10, *n* = 5; Sire 11, *n* = 5; Sire 12, *n* = 3; Sire 13, *n* = 2). Data were analyzed using one-way ANOVA and the Student-Newman-Keuls multiple comparison test. ^A–D^Means not sharing the same letter differ (*P* < 0.05)

An unpaired *t*-test analysis indicated that CIT-treated does had 13% greater daily milk production than CON-treated does (*P* $<$ 0.05) (Fig. 2). This study also revealed that sire affected milk production by does (*P* < 0.01) (Fig. 3).

### Milk production across days of lactation

**Fig. 4:**
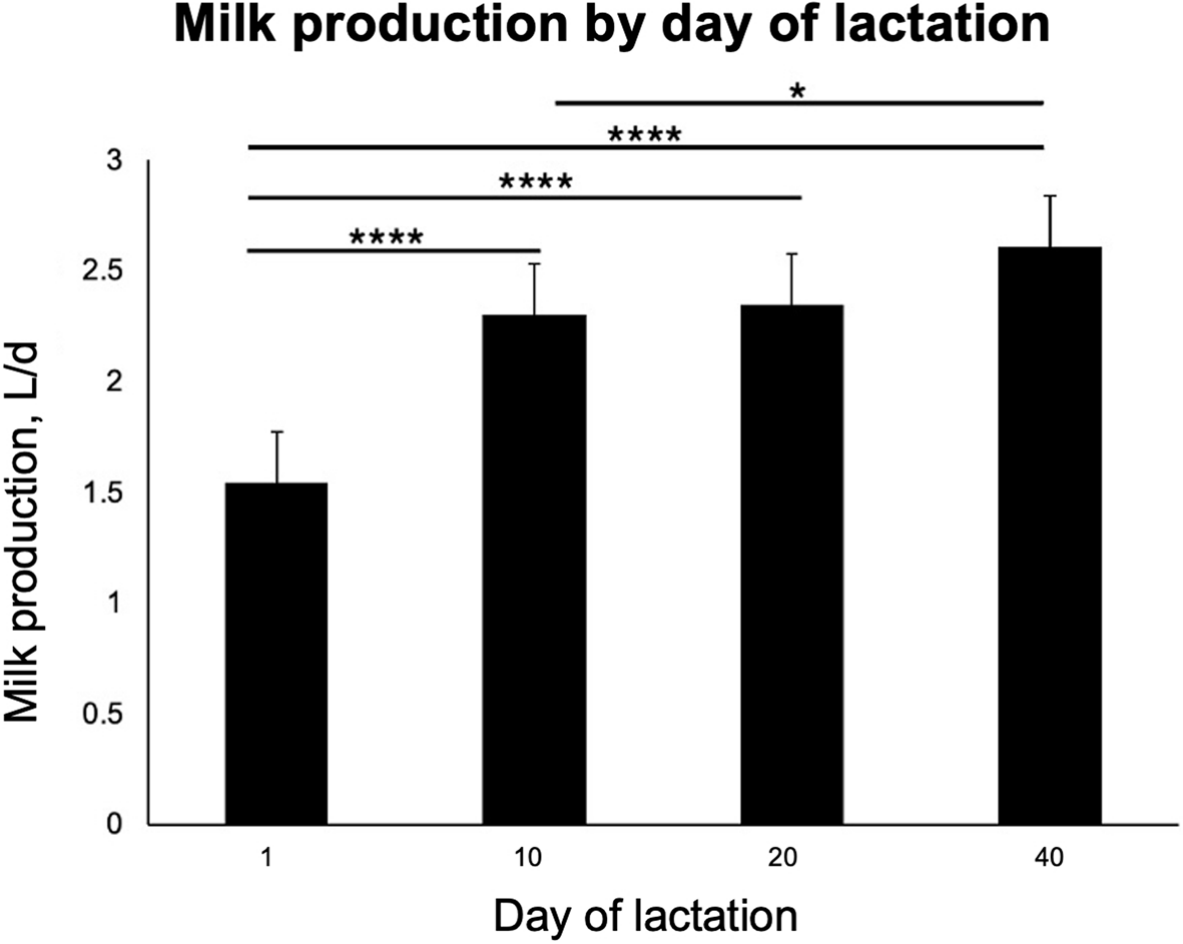
Daily milk production (L/d) by goats differed among days of lactation. Data were analyzed using ANOVA for a repeated-measures model and the Student-Newman-Keuls multiple comparison test [21]. Milk yield on d 40 of lactation was greater than that on d 0 (*n* = 47; *P* < 0.0001) and d 10 (*n* = 47; *P* < 0.05) of lactation. Milk production on d 20 of lactation was greater than that on d 0 (*P* < 0.0001) of lactation, and milk yield on d 10 of lactation was greater than that on d 0 of lactation (*P* < 0.0001). Asterisks indicate that means differ from one another (*P < 0.05, **P < 0.01, ***P < 0.001, ****P < 0.0001). Error bars represent SEM

Comparisons of milk production across days of lactation were assessed using the Wilcoxon/Kruskal-Wallis test that revealed an effect of day of lactation on milk production (*P* < 0.0001). A non-parametric pairwise comparison among days indicated that milk production on d 40 of lactation was greater than that on d 1 (*P* < 0.0001) and d 10 (*P* $<$ 0.05) of lactation. Milk production on d 20 of lactation was greater than that on d 1 (*P* < 0.0001) of lactation, and milk yield on d 10 of lactation was greater than that on d 1 of lactation (*P* < 0.0001). These results are presented in Fig. 4.

### Compositional analyses of milk

**Fig. 5:**
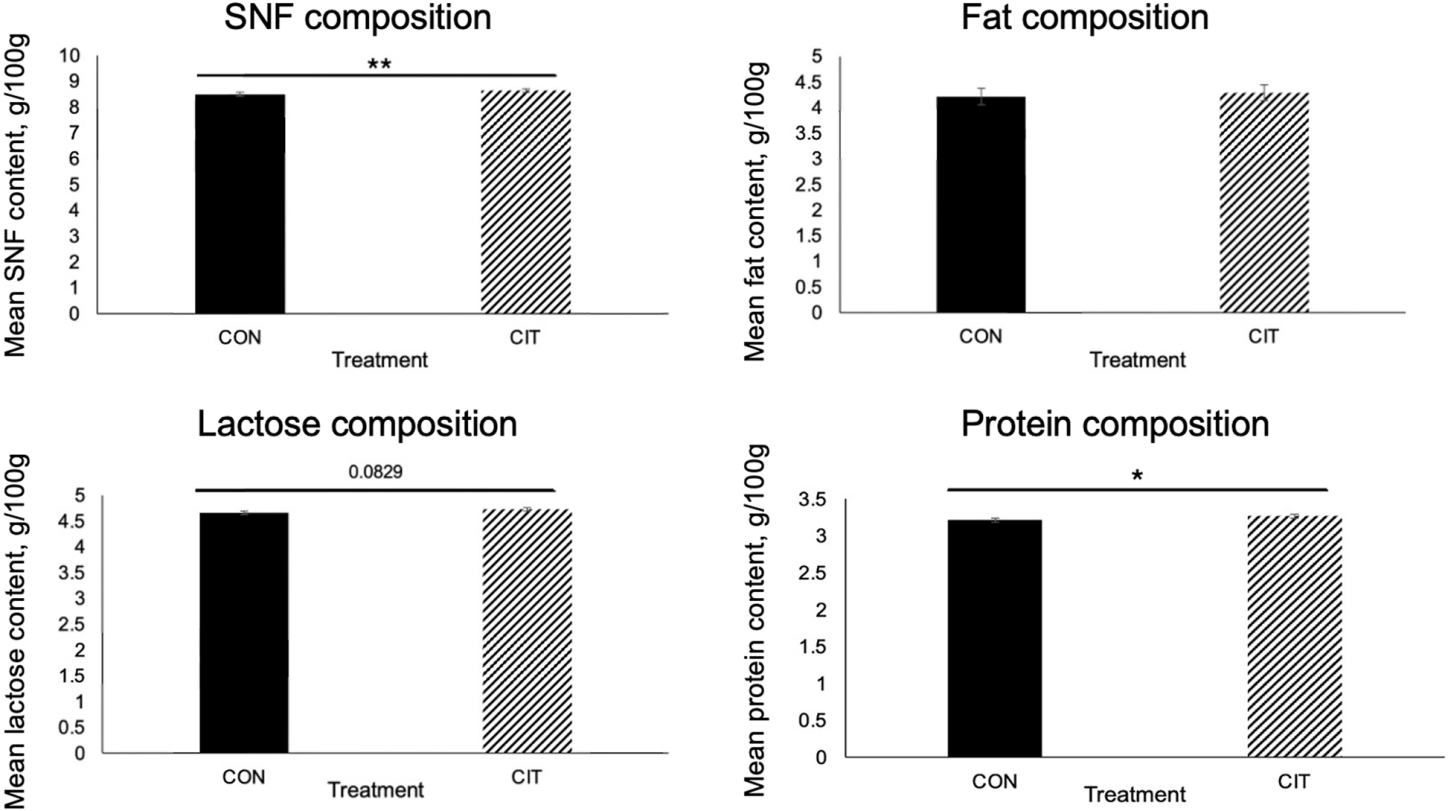
Composition of nutrients in the milk of goats fed diets supplemented with or without citrulline. The numbers of does are 24 and 23 for the CON and Cit groups, respectively. Data were analyzed using the unpaired *t*-test. Analyses of milk composition revealed that dietary supplementation of Cit increased solid-non-fat (SNF) content (*P* < 0.01) and protein content (*P* < 0.05) and tended to increase lactose content (*P* = 0.0829). No significant treatment effect on fat composition was detected. Asterisks indicate that means differ from one another (*P < 0.05, **P < 0.01). Error bars represent SEM

Concentrations of protein and Arg in milk collected on d 10, d 20, and d 40 of lactation were greater in the milk from CIT-treated than CON-treated does (*P* < 0.05), and there was a trend (*P* = 0.083) for greater lactose content in milk from CIT-treated than CON-treated does. Cit supplementation did not affect fat content in milk at any stage of lactation investigated (*P* > 0.05). Milk collected on d 10, 20, and 40 of lactation from CIT-treated does had greater concentrations of SNF than milk from CON-treated does (*P* < 0.01) throughout the lactation curve. Data on the composition of milk samples and their lactose, fat, protein, and SNF content are presented in Fig. 5.

### Concentrations of amino acids in serum

At the time of blood sampling, there was no significant effect (*P* > 0.05) of dietary Cit supplementation on concentrations of Arg, Cit, Orn, and alanine in serum (Table 2). The concentrations of aspartate, glutamate, asparagine, serine, glutamine, histidine, glycine, threonine, β-alanine, taurine, tyrosine, tryptophan, methionine, valine, phenylalanine, isoleucine, leucine, and lysine in serum of goats on d 0 and 30 of lactation were not affected (*P* > 0.05) by dietary supplementation of Cit, as shown in Table 3.

**Table 2:** Concentrations of citrulline, arginine, ornithine, and alanine in serum of lactating goats on d 0 and d 30 of lactation, nmol/mL

Item	Control (alanine supplementation)				Citrulline supplementation			
	**Cit**	**Arg**	**Orn**	**Ala**	**Cit**	**Arg**	**Orn**	**Ala**
Day 0	87 $\pm\ 49$	211 $\pm\ 42$	37 $\pm\ 26$	182 $\pm\ 46$	92 $\pm\ 42$	213 $\pm\ 29$	31 $\pm\ 11$	219 $\pm\ 65$
Day 30	184 $\pm\ 68$	217 $\pm\ 60$	74 $\pm\ 38$	220 $\pm\ 58$	150 $\pm\ 53$	219 $\pm\ 29$	65 $\pm\ 20$	239 $\pm\ 73$

Values are presented as mean ± SD. The numbers of does are 24 and 23 for the CON and Cit groups, respectively. Data were analyzed by ANOVA for a repeated-measures model [21]

**Table 3:** Concentrations of amino acids other than citrulline, arginine, ornithine and alanine in serum of goats on d 0 and d 30 of lactation, nmol/mL

Item	Control (alanine supplementation)				Citrulline supplementation			
	**Asp**	**Glu**	**Asn**	**Ser**	**Asp**	**Glu**	**Asn**	**Ser**
Day 0	1.8 $\pm\ 1.7$	120 $\pm\ 36$	41 $\pm\ 17$	90 $\pm\ 36$	2.7 $\pm\ 1.8$	131 $\pm\ 39$	38 $\pm\ 8$	62 $\pm\ 24$
Day 30	2.6 $\pm\ 1.9$	140 $\pm\ 35$	51 $\pm\ 18$	140 $\pm\ 39$	3.8 $\pm\ 1.9$	138 $\pm\ 42$	53 $\pm\ 18$	111 $\pm\ 31$
	**Gln**	**His**	**Gly**	**Thr**	**Gln**	**His**	**Gly**	**Thr**
Day 0	332 $\pm\ 77$	72 $\pm\ 14$	483 $\pm\ 162$	50 $\pm\ 21$	329 $\pm\ 53$	73 $\pm\ 13$	537 $\pm\ 163$	59 $\pm\ 23$
Day 30	349 $\pm\ 67$	57 $\pm\ 19$	$66\pm 167$	47 $\pm\ 23$	375 $\pm\ 104$	65 $\pm\ 11$	691 $\pm\ 272$	55 $\pm\ 29$
	**β-Ala**	**Tau**	**Tyr**	**Trp**	**β-Ala**	**Tau**	**Tyr**	**Trp**
Day 0	0.8 $\pm\ 0.2$	163 $\pm\ 37$	54 $\pm\ 25$	34 $\pm\ 13$	1.8 $\pm\ 0.8$	199 $\pm\ 49$	49 $\pm\ 8$	33 $\pm\ 9$
Day 30	1.1 $\pm\ 0.6$	149 $\pm\ 53$	60 $\pm\ 25$	35 $\pm\ 16$	1.7 $\pm\ 0.9$	134 $\pm\ 21$	55 $\pm\ 19$	38 $\pm\ 9$
	**Met**	**Val**	**Phe**	**Ile**	**Met**	**Val**	**Phe**	**Ile**
Day 0	34 $\pm\ 13$	165 $\pm\ 88$	47 $\pm\ 23$	117 $\pm\ 58$	32 $\pm\ 11$	178 $\pm\ 36$	50 $\pm\ 10$	106 $\pm\ 40$
Day 30	35 $\pm\ 16$	178 $\pm\ 35$	50 $\pm\ 11$	140 $\pm\ 71$	38 $\pm\ 9$	172 $\pm\ 54$	43 $\pm\ 12$	116 $\pm\ 36$
	**Leu**	**Lys**			**Leu**	**Lys**		
Day 0	161 $\pm\ 64$	138 $\pm\ 49$			151 $\pm\ 62$	122 $\pm\ 27$		
Day 30	140 $\pm\ 65$	156 $\pm\ 71$			169 $\pm\ 43$	145 $\pm\ 31$		

Values are presented as mean ± SD. The numbers of does are 24 and 23 for the CON and Cit groups, respectively. Data were analyzed by ANOVA for a repeated-measures model [21]

## Discussion

This study was designed to test the hypothesis that dietary supplementation of unencapsulated Cit would enhance lactational performance and milk composition as Cit would bypass the rumen and be converted to Arg, thereby increasing Arg availability and milk production. This hypothesis was formed based on results of previous studies demonstrating beneficial effects of Cit and Arg metabolism on reproductive performance and lactational performance in pigs [22, 23]. Similar to swine, the enterocytes of adult sheep have increased rates of intestinal synthesis of Cit and Arg from glutamate and Pro during pregnancy [17, 24]. Also, dietary supplementation with Cit enhances endogenous synthesis of Arg in cattle and sheep [14, 15]. In ruminants, Cit derived from the small intestine is locally converted into Arg via the intestinal-renal axis [17, 24]. In fed sheep, the small intestine releases Cit into the vasculature and the kidneys take up Cit at the rate of approximately 1.41 mmol/h [17]. Subsequently, the kidneys release Arg at approximately 1.46 mmol/h [17].

In ruminants, such as cows, goats, and sheep, virtually all dietary unencapsulated Arg is rapidly degraded in the rumen and does not reach the small intestine for absorption [17, 24]. However, extracellular Cit is not subject to microbial uptake and degradation in the rumen of cattle and sheep and can, therefore, be taken up in the small intestine for absorption [25]. Transporters for Cit, solute carrier family members SLC38A3 and SLC38A5, are not expressed by ruminal microbes so dietary Cit bypasses microbial degradation, unlike unprotected Arg which is rapidly degraded by ruminal microbes [26]. Cit transport is tissue- and cell-specific, and is carried out by transporters L, N, Β^0^, B^0,+^, and b^0,+^ [27]. Studies with sheep and cattle found no detectable uptake of ^14^C-labeled Cit by ruminal microbes, due to the lack of transporters, indicating that Cit may be supplemented without encapsulation to ruminants including goats and will not be degraded by ruminal microbes [16]. Thus, ewes fed a diet supplemented with Cit had greater concentrations of Cit, Orn, and Arg that increased linearly with increasing doses of Cit supplementation [28]. Concentrations of NO in serum were also 11.25% greater in the Cit-supplemented ewes compared to ewes fed a control diet [28]. Therefore, available results from studies in ruminants indicate that dietary Cit may be supplemented unencapsulated as a proxy for Arg as it is absorbed by the small intestine, then converted into Arg and utilized for the synthesis of NO, polyamines, creatine, and protein [17]. NO can enhance blood flow to lactating mammary glands and thus their uptake of AAs and other nutrients for polyamine-dependent milk synthesis [26]. In gestating gilts, dietary Cit supplementation from d 14 to 25 of gestation improved placental synthesis of NO and polyamines, as well as angiogenesis to improve embryonic development [23]. These results indicate that through dietary supplementation of Cit, the endogenous synthesis of Arg was increased for the enhancement of synthesis of NO and polyamines in goats as reported for swine [23].

In our current study, dietary supplementation of unencapsulated Cit increased daily milk production and milk protein content in comparison to CON-treated does, which supports our hypothesis. Higher concentrations of protein in milk from Cit-supplemented goats also aligned with the hypothesis that an increase in the availability of Arg may serve as a building block of polypeptide, activate the mechanistic target of rapamycin cell signaling pathway, and increase polyamine production that would increase the synthesis of milk proteins by the mammary glands of the lactating goats. Arguably, this effect was not coordinate with increases in concentrations of Cit and Arg in the serum of the Cit group at the time of blood sampling, but the effect could result from more efficient transfer of available AAs from maternal blood to the mammary glands, as indicated by the increased output of protein in milk (Fig. 5). In order to address this dilemma, future experiments may determine differences in concentrations of Arg and other amino acids in arterial blood to the mammary gland and venous blood draining the mammary gland in order to calculate net uptake of amino acids by the mammary gland. There were no significant interactions between treatment and litter size, parity, day of lactation, or sire; therefore, effects of dietary Cit supplementation were independent of those factors.

There was a main effect of litter size on milk production (*P* $<$ 0.05) with an increase in litter size resulting in increased milk production as expected [29]. An increase in litter size would increase placental mass and, in turn, increase lactogenic hormones such as placental lactogen that is associated with alveolar development in the mammary glands during pregnancy [29]. That study also revealed a significant effect of litter size on daily milk production as expected [29]. Parity of does also affected (*P* $<$ 0.01) milk production, which is consistent with reports that ewes experiencing 2 and 3 parities produced more milk than primiparous ewes [30]. Our present study utilized 13 alpine bucks and there was a significant effect of sire on milk production (*P* $<$ 0.05). This has also been reported to be an effect of sire of fetus on milk yield in cattle [31]. Collectively, novel results of this study support our hypothesis that dietary supplementation of unprotected Cit can increase Arg synthesis by extra-hepatic tissues, daily milk production, and the concentrations of protein and SNF in milk from lactating Alpine dairy goats.

Results of this study have important implications for increasing the economic returns of goat production. The current price of goat milk in a typical U.S. food store is $6.26/L [32]. The milk yield was 2.06 and 2.33 L/d per goat in the CON and Cit groups, respectively (Fig. 2). An increase of 0.27 L milk/d would result in an additional profit of $1.69/d from a dairy goat. The current price of Cit on the global market is $8/kg [33]. The cost of 11.5-g Cit/d per goat is only $0.09 and the net benefit of Cit supplementation is $1.6/d per goat. Thus, a 13% increase in milk production due to dietary Cit supplementation amounts to $16/d or $44,800 during a 280-d lactation period for a farm with 100 dairy goats.

## Conclusions

The results of this study support our original hypothesis that dietary supplementation with unencapsulated Cit would enhance lactational performance and effect milk composition. Dietary supplementation of Cit increased SNF and protein (including Arg) content of goat milk and tended to increase lactose content in the milk. In this study, effects of day of lactation, litter size, parity, and sire were all consistent with effects reported for other species with each having a significant effect on daily milk production. As no significant interactions between treatment and litter size, parity, day of lactation, or sire were revealed in this study, indicates that the effects of dietary Cit supplementation were independent of those factors. These results reveal beneficial management strategies which could improve production, efficiency, and overall profits in the dairy goat industry. This study was limited to the use of does that were group fed ad-libitum either the control diet or Cit-supplemented diet which is how this management strategy would be practiced on a commercial dairy goat enterprise and as a common practice in other livestock production programs with ruminants. Collectively, the novel results of this study revealed that dietary supplementation of unencapsulated Cit fed ad-libitum may be utilized to enhance lactational performance and increase milk SNF and protein content in goats.

## Data Availability

The datasets used and/or analyzed during the current study are presented and available from the corresponding author upon reasonable request.

## Abbreviations

AA: Amino acid
Arg: Arginine
Cit: Citrulline
HPLC: High performance liquid chromatography
NO: Nitric oxide
OPA: *o*-Phthaldialdehyde
SNF: Solid-non-fat
